# The complete mitochondrial genome of the threatened Neotropical catfish *Lophiosilurus alexandri* (Silurifomes: Pseudopimelodidae) and phylogenomic analysis indicate monophyly of Pimelodoidea

**DOI:** 10.1590/1678-4685-GMB-2016-0007

**Published:** 2016-09-19

**Authors:** Daniel Cardoso Carvalho, Violeta da Rocha Perini, Alex Schomaker Bastos, Igor Rodrigues da Costa, Ronald Kennedy Luz, Carolina Furtado, Francisco Prosdocimi

**Affiliations:** 1 Laboratório de Genética da Conservação, Programa de Pós-Graduação em Biologia dos Vertebrados, Pontifícia Universidade Católica de Minas Gerais (PUC Minas), Belo Horizonte, MG, Brazil.; 2 Laboratório de Genômica e Biodiversidade, Instituto de Bioquímica Médica Leopoldo de Meis, Universidade Federal do Rio de Janeiro (UFRJ), Rio de Janeiro, RJ, Brazil.; 3 Laboratório de Aquacultura da Escola de Veterinária da Universidade Federal de Minas Gerais (UFMG, Belo Horizonte, MG, Brazil.; 4 Instituto Nacional do Câncer (INCA), Rio de Janeiro, RJ, Brazil.

**Keywords:** Mitogenome, fish, next-generation-sequencing, Illumina, Pseudopimelodidae

## Abstract

*Lophiosilurus alexandri* is an endemic catfish from the São Francisco River Basin (Brazil) popularly known as pacamã, which has economic potential for aquaculture farming. The mitochondrial genome was sequenced for the threatened Neotropical catfish *L. alexandri*. Assembly into scaffolds using MIRA and MITObim software produced the whole, circularized mitochondrial genome, which comprises 16,445 bp and presents the typical gene arrangement of Teleostei mitochondria. A phylogenomic analysis was performed after the concatenation of all proteins obtained from whole mitogenomes of 20 Siluriformes and two outgroups. The results confirmed the monophyly of nine families of catfishes and also clustered *L. alexandri* as a sister group to the family Pimelodidae, thus confirming the monophyly of the superfamily Pimelodoidea. This is the first mitochondrial phylogenomics study for Pimelodoidea and the first mitogenome described for the Pseudopimelodidae family, representing an important resource for phylogeography, evolutionary biology, and conservation genetics studies in Neotropical fishes.

Catfishes (Siluriformes) are a highly diverse order composed of 39 families and more than 3,700 living species (Eschmeyer and Fong, 2016). The fish *Lophiosilurus alexandri* (Steindachner, 1877), popularly known as pacamã, is an endemic catfish from the São Francisco River Basin (Brazil) and belongs to the Pseudopimelodidae family, a taxon broadly distributed in South America (Eschmeyer and Fong, 2016). *L. alexandri* is a carnivorous species that displays parental care and has economic potential for aquaculture farming (Sato *et al.*, 2003; dos Santos and Luz, 2009).

Pacamã may be cultivated in captivity after adapting broodstock to aquaculture conditions (Costa *et al.*, 2015), however its natural populations have declined over the last decades. Threats such as overfishing and environmental degradation led to the inclusion of *L. alexandri* in the Brazilian red list of threatened species (Brasil, 2014).

Here we present the complete, circularized version of the whole mitochondrial genome and the phylogenomic relationships of pacamã to 20 other related Siluriformes and two outgroups using a supermatrix approach.

Muscle fragments were obtained from a freshly captured *L. alexandri*. The voucher specimen was fixed in 10% formalin and later preserved in 70% ethanol. (voucher: LGC6088 at PUC Minas Natural History Museum). Genomic DNA was extracted using a modified salting-out method (Sunnucks and Hales, 1996) and nebulized for 6 min to obtain 200-600 bp fragments.

Partial genome sequencing was carried out using a Nextera kit in a sixth of an Illumina HiSeq 2000 lane. An initial mitogenome assembly generated by MIRA was used as an input for the MITObim algorithm (Hahn *et al.*, 2013) using default parameters. Mitogenome coverage was obtained using Tablet software (Milne *et al.*, 2013). Mitos WebServer (Bernt *et al.*, 2013) and MitoFish (Iwasaki *et al.*, 2013) were used for annotation. Blast searches (Altschul *et al.*, 1997) against fish amino acid sequences confirmed gene boundaries. tRNA predictions were confirmed using tRNAscan-SE (Lowe and Eddy, 1997). Ribosomal RNA annotations were estimated through automatic analysis provided by Mitos Web Server and MitoFish followed by visual inspection of nucleotide sequence alignments against other Pimelodidae rRNAs.

The complete mitochondrial genome for *L. alexandri* was assembled using 0.06% (203,036 reads) of the total paired-end reads sequenced (33,839,478 reads of 100 bp each). The assembly provided a circular mitogenome with 134.1 x coverage comprised 16,445 bp, a size similar to the average mitogenome of catfishes. Its gene content also followed the typical pattern for teleost mitogenomes (Prosdocimi *et al.*, 2012, Song *et al.*, 2012, Zhang *et al.*, 2013, Perini *et al.*, 2014), being composed of 37 genes, including 13 protein-coding genes, 22 tRNAs, 2 rRNAs, and 1 non-coding control region (Table S1, Supplementary Material). The mitogenome of *Lophiosilurus alexandri* is available in GenBank under the accession number KJ494387.

An in-house pipeline developed in Python (https://github.com/igorrcosta/phylomito) was used to: (i) concatenate individual alignments of mitochondrial proteins, (ii) retrotranslate these alignments into codons/nucleotides, and (iii) provide a supermatrix dataset that has been used as input into MEGA 7 for modeltest and phylogeny reconstruction (Kumar *et al.*, 2016).

The phylogenetic relationship between *L. alexandri* and the other 20 catfishes and two outgroups from the Gymnotiformes and Characiformes orders (Table 1) was recovered using a supermatrix approach of 11,468 nucleotides produced after the concatenation and reverse translation of protein sequence alignments for all the 13 mitochondrial proteins. A maximum likelihood tree was generated using all alignment sites with the best model found by MEGA (GTR+G+I). The phylogenomic analysis produced a consistent tree in accordance with phylogenetic evidence obtained using rag1 and rag2 nuclear genes recovered using 3,660 base pairs (Sullivan *et al.*, 2006). Whole mitochondrial data, however, also evidenced new features, such as (i) a more ancestral split of Amblycipitidae species and also (ii) a highly supported clade (bootstrap=93) grouping families Ictaluridae, Cranogladidae and Pangasidae. *L. alexandri, Pimelodus pictus* and two species from the genus *Pseudoplatystoma* were clustered in the same clade with a very confidence support after 1000 resamplings (bootstrap=100) (Figure 1), corroborating the monophyletic relationship of the Pimelodoidea clade, as previously recovered elsewhere (Sullivan *et al.*, 2006). The mitogenome described here is the first representative for the family Pseudopimelodidae.

**Table 1:** List of species, taxonomic information and accession numbers used in the phylogenetic analyses.

Order	Family	Species	GenBank ID	Size (bp)	Reference
Siluriformes	Doradidae	*Amblydoras gonzalezi*	NC_015745.1	16505	Nakatani *et al*., 2011
Siluriformes	Doradidae	*Platydoras armatulus*	NC_025585.1	16470	Liu *et al*., 2016
Siluriformes	Cranoglanididae	*Cranoglanis bouderius*	NC_008280.1	16539	Peng *et al*., 2006
Siluriformes	Clariidae	*Clarias sp.*	NC_015749.1	16508	Nakatani *et al*., 2011
Siluriformes	Clariidae	*Clarias fuscus*	NC_023924.1	16518	Zhou *et al*., 2015
Siluriformes	Ictaluridae	*Ictalurus punctatus*	NC_003489.1	16497	Waldbieser *et al*., 2003
Siluriformes	Ictaluridae	*Ictalurus furcatus*	NC_028151.1	16499	Liu *et al.* (Unpublished)
Siluriformes	Amblycipitidae	*Liobagrus marginalis*	NC_022923.1	16483	Li *et al*., 2014
Siluriformes	Amblycipitidae	*Liobagrus nigricauda*	NC_021407.1	16512	Jia *et al*., 2013b
Siluriformes	Amblycipitidae	*Liobagrus obesus*	NC_008232.1	16531	Kartavtsev *et al*., 2007
Siluriformes	Pangasiidae	*Pangasianodon gigas*	NC_006381.1	16533	Jondeung *et al*., 2007
Siluriformes	Pangasiidae	*Pangasianodon hypophthalmus*	NC_021752.1	16522	Zhao *et al*., 2014
Siluriformes	Pangasiidae	*Pangasius larnaudii*	NC_015839.1	16471	Nakatani *et al*., 2011
Siluriformes	Pangasiidae	*Pangasius pangasius*	NC_023924.1	16476	Mohindra *et al*., 2015
Siluriformes	Siluridae	*Silurus glanis*	NC_014261.1	16526	Vitta *et al*., 2011
Siluriformes	Siluridae	*Silurus meridionalis*	NC_014866.1	16527	Liang *et al*., unpublished
Siluriformes	Siluridae	*Pterocryptis conchichinensis*	NC_027107.1	16501	Xu *et al*., 2016
Siluriformes	Pseudopimelodidae	*Lophiosilurus alexandri*	KJ494387	16445	Present work
Siluriformes	Pimelodidae	*Pimelodus pictus*	NC_015797.1	16575	Nakatani *et al*., 2011
Siluriformes	Pimelodidae	*Pseudoplatystoma corruscans*	NC_026846.1	16123	Prosdocimi *et al.* (Unpublished)
Siluriformes	Pimelodidae	*Pseudoplatystoma magdaleniatum*	NC_026526.1	16568	Rangel-Medrano *et al*., 2015
Characiformes	Characidae	*Paracheirodon axelrodi*	NC_023270.1	17100	Zhang *et al*., 2014
Gymnotiformes	Hypopomidae	*Brachyhypopomus occidentalis*	NC_015078.1	16542	Lavoue *et al*., 2012

**Figure 1:**
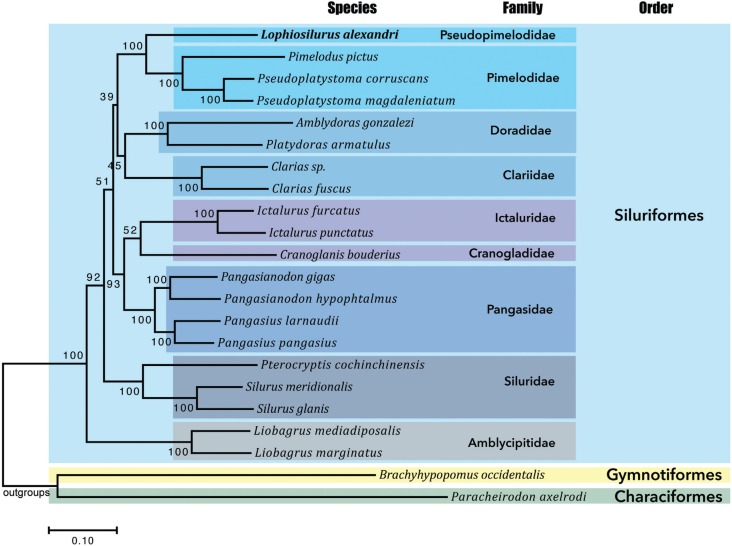
Molecular phylogenetic analysis of pacamã, 20 catfishes and two outgroups. A total of 11,468 positions were analyzed consisting in the concatenation of 13 mitochondrial genes. A tree was built using maximum likelihood approaches with GTR+G+I model. Outgroups were used to root the tree. All families and genera of catfishes analyzed were revealed as monophyletic (colored boxes). Bootstrap values (1000 replicates) are shown at the corresponding nodes.

## Supplementary Material

The following online material is available for this article:

Table S1 - Mitochondrial genome characteristics of *L. alexandri* (KJ494387).

## References

[B1] Altschul SF, Madden TL, Schäffer AA, Zhang J, Zhang Z, Miller W, Lipman DJ (1997). Gapped BLAST and PSI-BLAST: A new generation of protein database
search programs. Nucleic Acids Res.

[B2] Bernt M, Donath A, Jühling F, Externbrink F, Florentz C, Fritzsch G, Pütz J, Middendorf M, Stadler PF (2013). MITOS: Improved de novo metazoan mitochondrial genome
annotation. Mol Phylogenet Evol.

[B3] Brasil (2014). Lista Nacional Oficial de Espécies da Fauna Ameaçadas de Extinção - Peixes e
Invertebrados Aquáticos. Portaria MMA n° 445.

[B4] Costa DC, Silva WDS, Melillo R, Miranda KC, dos Santos JCE, Luz RK (2015). Capture, adaptation and artificial control of reproduction of
*Lophiosilurus alexandri* A carnivorous freshwater
species. Anim Reprod Sci.

[B5] dos Santos JCE, Luz RK (2009). Effect of salinity and prey concentrations on *Pseudoplatystoma
corruscans Prochilodus costatus* and *Lophiosilurus
alexandri* larviculture. Aquaculture.

[B6] Hahn C, Bachmann L, Chevreux B (2013). Reconstructing mitochondrial genomes directly from genomic
next-generation sequencing reads - A baiting and iterative mapping
approach. Nucleic Acids Res.

[B7] Iwasaki W, Fukunaga T, Isagozawa R, Yamada K, Maeda Y, Satoh TP, Sado T, Mabuchi K, Takeshima H, Miya M (2013). MitoFish and MitoAnnotator: A mitochondrial genome database of fish
with an accurate and automatic annotation pipeline. Mol Biol Evol.

[B8] Kumar S, Stecher G, Tamura K (2016). MEGA7: Molecular Evolutionary Genetics Analysis version 7.0 for bigger
datasets. Mol Biol Evol.

[B9] Li Q, Du J, Liu Y, Zhou J, Ke H, Liu C, Liu G (2014). The complete mitochondrial genome of *Liobagrus
marginatus* (Teleostei, Siluriformes: Amblycipitidae). Mitochondrial DNA.

[B10] Liu S, Yao J, Zhang J, Liu Z (2016). Next generation sequencing yields the complete mitochondrial genome of
the striped raphael catfish, *Platydoras armatulus* (Siluriformes:
Doradidae). Mitochondrial DNA.

[B11] Lowe TM, Eddy SR (1997). tRNAscan-SE: A program for improved detection of transfer RNA genes in
genomic sequence. Nucleic Acids Res.

[B12] Milne I, Stephen G, Bayer M, Cock PJA, Pritchard L, Cardle L, Shaw PD, Marshall D (2013). Using Tablet for visual exploration of second-generation sequencing
data. Brief Bioinform.

[B13] Mohindra V, Singh RK, Kumar R, Sah RS, Lal KK (2015). Complete mitochondrial genome sequences of two endangered Indian
catfish species, *Clarias batrachus* and *Pangasius
pangasius*. Mitochondrial DNA.

[B14] Nakatani M, Miya M, Mabuchi K, Saitoh K, Nishida M (2011). Evolutionary history of Otophysi (Teleostei), a major clade of the
modern freshwater fishes: Pangaean origin and Mesozoic radiation. BMC Evol Biol.

[B15] Perini VDR, Carvalho DC, Beheregaray LB, Prosdocimi F (2014). The complete mitochondrial genome of the southern purple-spotted
gudgeon *Mogurnda adspersa* (Perciformes: Eleotridae) through
pyrosequencing. Mitochondrial DNA.

[B16] Prosdocimi F, Carvalho DC, Almeida RN, Beheregaray LB (2012). The complete mitochondrial genome of two recently derived species of
the fish genus *Nannoperca* (Perciformes,
Percichthyidae). Mol Biol Rep.

[B17] Rangel-Medrano JD, Alzate JF, Márquez EJ (2015). Complete mitochondrial genome of the Neotropical catfish
*Pseudoplatystoma magdaleniatum* (Siluriformes,
Pimelodidae). Mitochondrial DNA.

[B18] Sato Y, Fenerich-Verani N, Godinho HP, Godinho HP, Godinho AL (2003). Induced reproduction of fishes of the São
Francisco. Waters, Fishes, and Fishermen of the São Francisco of Minas Gerais.

[B19] Song HY, Satoh TP, Mabuchi K (2012). Complete mitochondrial genome sequence of the dragonet
*Callionymus curvicornis* (Perciformes: Callionymoidei:
Callionymidae). Mitochondrial DNA.

[B20] Sullivan JP, Lundberg JG, Hardman M (2006). A phylogenetic analysis of the major groups of catfishes (Teleostei:
Siluriformes) using rag1 and rag2 nuclear gene sequences. Mol Phylogenet Evol.

[B21] Sunnucks P, Hales D. (1996). Numerous transposed sequences of mitochondrial cytochrome oxidase I-II
in aphids of the genus *Sitobion* (Hemiptera:
aphididae). Mol Biol Evol.

[B22] Xu J, Han C, Huang JR (2016). The complete mitogenome of the sheatfish *Pterocryptis
cochinchinensis* (Siluriformes: Siluridae) and phylogenetic
implications. Mitochondr ial DNA.

[B23] Zhang Z, Zhao L, Song N, Gao T (2013). The complete mitochondrial genome of *Johnius grypotus*
(Perciformes: Sciaenidae). Mitochondrial DNA.

[B24] Zhou C, Wang X, Guan L, He S (2015). The complete mitochondrial genome of *Clarias fuscus*
(Teleostei, Siluriformes: Clariidae). Mitochondrial DNA.

