# Supplementary material for: The complete mitochondrial genome of the threatened Neotropical catfish Lophiosilurus alexandri (Silurifomes: Pseudopimelodidae) and phylogenomic analysis indicate monophyly of Pimelodoidea
Source: Genet Mol Biol. 2016 Sep 19;39(4):674–7. doi: 10.1590/1678-4685-GMB-2016-0007 (PMC5127154; doi:10.1590/1678-4685-GMB-2016-0007)
Supplement: Supplementary file 1 [file 1415-4757-gmb-1678-4685-GMB-2016-0007-Suppl01.pdf]

Table S1 - Mitochondrial genome characteristics of *L. alexandri* (KJ494387).

Gene	Position		Size		Codon		Intergenic	
	Start	End	Nucleotides	Amino acids	Start	Stop	space (bp)	Strand
tRNA(Phe)	1	68	68				0	H
12S rRNA	69	1034	966				22	H
tRNA(Val)	1057	1128	72				0	H
16S rRNA	1129	2798	1670				-2	H
tRNA(Leu)	2797	2871	75				1	H
ND1	2873	3841	969	323	ATG	TAA	5	H
tRNA(Ile)	3847	3918	72				-1	H
tRNA(Gln)	3918	3988	71				-1	L
tRNA(Met)	3988	4057	70				0	H
ND2	4058	5101	1044	348	ATG	T-	1	H
tRNA(Trp)	5103	5183	81				10	H
tRNA(Ala)	5194	5262	69				1	L
tRNA(Asn)	5264	5336	73				30	L
tRNA(Cys)	5367	5432	66				1	L
tRNA(Tyr)	5434	5503	70				1	L
COX1	5505	7052	1548	516	GTG	TAA	4	H
tRNA(Ser)	7057	7127	71				4	L
tRNA(Asp)	7132	7202	71				14	H
COX2	7217	7906	690	230	ATG	T-	1	H
tRNA(Lys)	7908	7981	74				1	H
ATP8	7983	8147	165	55	ATG	TAA	-7	H
ATP6	8141	8821	681	227	ATG	TA-	2	H
COX3	8824	9606	783	261	ATG	T-	1	H
tRNA(Gly)	9608	9680	73				0	H
ND3	9681	10028	348	116	ATG	T-	1	H
tRNA(Arg)	10030	10098	69				0	H
ND4L	10099	10392	294	98	ATG	TAA	-4	H
ND4	10389	11768	1380	460	ATG	T-	1	H
tRNA(His)	11770	11839	70				0	H
tRNA(Ser)	11840	11906	67				6	H
tRNA(Leu)	11913	11985	73				0	H
ND5	11986	13806	1821	607	ATG	TAA	2	H
ND6	13809	14321	513	171	ATG	TA-	0	L
tRNA(Glu)	14322	14390	69				1	L
CYTB	14392	15525	1134	378	ATG	T-	1	H
tRNA(Thr)	15527	15598	72				-2	H
tRNA(Pro)	15597	15666	70				0	L
Control region	15667	16445	779					
